# The attitudes of individuals with or at risk of adult‐onset genetic conditions on reproductive genetic testing: A systematic review

**DOI:** 10.1002/jgc4.70079

**Published:** 2025-07-01

**Authors:** Shanice Allen, Alisdair McNeill, Christopher McDermott, Felicity Boardman, Jade Howard

**Affiliations:** ^1^ Division of Neuroscience & Neuroscience Institute The University of Sheffield Sheffield UK; ^2^ Sheffield Clinical Genetics Department Sheffield Childrens Hospital NHS Foundation Trust Sheffield UK; ^3^ Academic Directorate of Neuroscience Sheffield Teaching Hospitals NHS Foundation Trust Sheffield UK; ^4^ University of Warwick Coventry UK

**Keywords:** decision‐making, genetic diseases, late‐onset diseases, preimplantation genetic testing, prenatal testing, reproductive genetic testing, reproductive options

## Abstract

Individuals who carry a genetic variant for a genetic disease can access reproductive genetic testing in order to prevent the transmission of the gene variant to their children. This systematic review aimed to synthesize the findings from both qualitative and quantitative literature to understand these individuals' attitudes toward pre‐implantation genetic testing (PGT) and prenatal testing (PNT) and how they make decisions around them. A systematic search was undertaken following PRISMA guidelines, with 37 articles meeting the inclusion criteria for evaluating experiences and attitudes of individuals with or at risk of adult‐onset genetic conditions on reproductive genetic testing. Relevant findings from each study were included in a thematic synthesis. Five analytical themes were generated to elucidate the attitudes toward reproductive genetic testing and the factors that impact decision‐making in individuals with or at risk of late‐onset genetic diseases: (1) Preventing gene transmission; (2) finding the threshold: evaluating the necessity of reproductive genetic testing; (3) ethical/acceptability considerations; (4) external influences in decision‐making; and (5) psychological and practical concerns of reproductive genetic testing. This review highlights several factors that influence attitudes toward reproductive genetic testing. Complex decision‐making was a cross‐cutting experience that characterizes and defines reproductive genetic testing for late‐onset conditions. There was a general consensus of support for reproductive genetic testing and a belief that it should be available to all. The need for awareness and education on reproductive genetic testing is evident. Future work should look at how to address these knowledge deficits, while exploring individuals' preferences for when and by whom information is delivered. Acknowledging the complexity of decision‐making can encourage meaningful discussions and address potential issues.

**What is known about this topic**

Previous reviews have examined attitudes toward PGT specifically (with less focus on PNT) in those with early‐onset genetic disorders and some hereditary cancers.

**What this paper adds to the topic**

This is the first review to explore the acceptability and attitudes of individuals with or at risk of adult‐onset genetic conditions regarding reproductive genetic testing, including both pre‐implantation genetic testing and prenatal testing, and including both quantitative and qualitative studies. It identifies five key themes which expand the understanding of reproductive decision‐making and highlight the complexities of decision‐making, offering insights for future research.

## 1 INTRODUCTION

Individuals who carry a genetic variant for a genetic disease can access reproductive genetic testing in order to prevent the transmission of the gene variant to their children. Reproductive genetic testing consists of prenatal testing (PNT) and preimplantation genetic testing (PGT) (formally prenatal diagnosis and preimplantation genetic diagnosis). PNT involves testing a pregnancy to see if the fetus carries the genetic variant. Individuals that utilize PNT typically do so with the intention of terminating an affected pregnancy. Chorionic villus sampling (CVS) and amniocentesis are two techniques in PNT. Amniocentesis involves using a very thin needle to extract a small amount of amniotic fluid. CVS involves taking a tissue sample (chorionic villi) from the placenta by aspiration through a transcervical catheter or transabdominal needle. PGT is an alternative to PNT where the genetic test is performed on embryos before the pregnancy is established. Embryos are obtained via in vitro fertilization (IVF), genetically tested at the blastocyst stage for the disease‐causing genetic variants, and only unaffected embryos are transferred into the uterus (Basille et al., 2009). In situations where an individual does not want to know their own genetic status but still wishes to avoid passing on a genetic variant to their child, then PNT and PGT can be carried out using exclusion or non‐disclosure methods (ePNT/ePGT). In these approaches, DNA samples are taken from a family member known to carry the genetic condition—usually the parent of the at‐risk individual (i.e., the grandparent of the potential child). The embryo or fetus is then tested to determine whether it has inherited the segment of DNA from the affected grandparent. If it has, this indicates a 50% chance of the fetus/embryo carrying the same risk as the at‐risk parent. However, a key limitation of this method is that it does not directly test for the specific genetic variant. Instead, it only assesses whether the embryo or fetus has inherited a stretch of DNA from the affected side of the family. As a result, unaffected embryos or pregnancies might be discarded or terminated, especially if the at‐risk parent has not actually inherited the mutation themselves. This indirect method is used to protect the at‐risk individual's right not to know their genetic status (Braude et al., 1998; Millan et al., 1989). Other reproductive options including gamete donation, adoption, and the option of not having children exist alongside these; however, this review focuses on attitudes toward reproductive genetic testing which enables individuals to have biological children without the genetic variant.

Individuals with or at risk of genetic diseases often struggle to make decisions on reproductive genetic testing (Boardman & Hale, 2018; Genoff Garzon et al., 2018; Redgrave & McNeill, 2022). It can be viewed as more controversial to use reproductive genetic testing for diseases that are late onset, such as hereditary breast and ovarian cancer (HBOC)/Huntington's disease (HD) than for early‐onset genetic diseases, that is, cystic fibrosis (CF), chromosomal disorders, or Duchenne muscular dystrophy (DMD) due to individuals with these disease‐causing genetic variants being able to live healthy lives for years before the disease becomes an active concern (Ethics Committee of the American Society for Reproductive Medicine, 2013).

Previous reviews have examined attitudes toward PGT in those with genetic disorders (Cunningham et al., 2015; Hershberger & Pierce, 2010; Hughes et al., 2021) and hereditary cancers (Lombardi et al., 2022; Quinn et al., 2012). However, this is the first review to explore the acceptability and attitudes of individuals with or at risk of adult‐onset genetic conditions regarding PNT/PGT, including both quantitative and qualitative studies. It expands understanding of reproductive genetic testing and decision‐making, offering insights for future research.

## 2 METHODS

The study was registered in the international prospective register of systematic reviews (PROSPERO #CRD42023487632).

### 2.1 Search strategy

Articles were systematically searched on Web of Science and Scopus (February 15, 2024), with the full search strategy in Table 1. Reference lists of the included articles were also manually searched via forward citation searching.

**TABLE 1:** Search strategy.

	Main concepts		Alternative terms
	View*	OR	experience* or attitude* or opinion* or knowledge
AND	Reproductive technique*	OR	“Reproductive options” or “preimplantation genetic diagnosis” or “prenatal diagnosis” or “preimplantation genetic testing” or “prenatal testing” or PGT or PNT or PND or PGD or amniocentesis or “chorionic villus sampling” or cvs
AND	Adult‐onset genetic disease	OR	“Breast neoplasm*” or “Breast cancer” or BRCA1 or BRCA2 or “ovarian cancer” or “ovarian neoplasm*” or “prostate cancer” or “bowel cancer” or “bowel neoplasm*” or “pancreatic cancer” or “pancreatic neoplasm*” or “Lynch syndrome” or “hereditary non polyposis colon cancer” or “HNPCC” or MLH1 or MSH2 or MSH6 or PMS2 or “Li‐Fraumeni syndrome” or “Cowden syndrome” or “PTEN Hamartoma tumour syndrome” or “Familial adenomatous polyposis” or FAP or APC or “Peutz Jeghers syndrome” or PJS or STK11 or BMPR1A or SMAD4 or PALB2 or “Von Hippel Lindau syndrome” or VHL or “Birt‐Hogg‐Dube syndrome” or BHDS or “Multiple endocrine neoplasia” or MEN1 or MEN2 or “Familial atypical multiple mole melanoma syndrome” or FAMMM or “Hereditary papillary cancer” or HPRCC or HLRCC or cancer or “neurodegenerative disease*” or “huntington*” or HD or “prion disease” or “motor neuron disease” or MND or ALS or “amyotrophic lateral sclerosis” or alzheimers or dementia or “frontotemporal dementia” or FTD or “Charcot–Marie–Tooth disease” or CMT or “Myotonic dystrophy” or DM or “Spinocerebellar ataxia” or SCA or “hereditary cardiovascular disease*” or “hypertrophic cardiomyopathy” or HCM or “Hereditary Long‐QT syndrome” or LQTS

### 2.2 Study selection

Peer‐reviewed articles in English published from 1997 onward were included if they examined motivations, attitudes, decision‐making, and experiences with reproductive genetic testing in individuals with genetic variants or at risk of adult‐onset hereditary diseases (defined as conditions presenting from age 16 onward). The search was limited to papers published on or after 1997 (i.e., the establishment of the ESHRE PGT Consortium, which reports annual PGT data, and when the first PGT service on the NHS in the United Kingdom was offered (Harper et al., 2012; NHS Choices, 2024)). Full inclusion/exclusion criteria are in Table 2.

**TABLE 2:** The inclusion and exclusion criteria.

Inclusion criteria	Exclusion criteria
Studies had to report on primary research	Reviews of the literature
Studies had to be published in a peer‐reviewed journal	Unpublished studies
Studies had to be written in English	Studies not in English
Full article available	Full article not available
Published in 1997or later	Published before 1997
Studies had to address the decision‐making process about at least one reproductive technique	Studies that took into account only test uptake without analysis of the reasons for accepting or declining tests or implemented with decision support tools
Disease must be adult onset	Studies investigating early‐onset diseases such as aneuploidy screening (PGT‐A), structural chromosome rearrangement (PGT‐SR), or down syndrome
Participants must be over 18 with gene variant of disease or at genetic risk of developing disease	Studies involving participants under 18
Studies including the views of people with or at risk toward reproductive genetic testing	Studies focusing on healthcare professionals' attitudes toward reproductive genetic testing

### 2.3 Study screening

Duplicates were removed through Zotero (6.0.30), and studies were screened following PRISMA guidelines (Page et al., 2021) by title, abstract, and full text, including reference lists. A hierarchical abstract screening tool was created for efficiency (Polanin et al., 2019). To ensure reliability, a second reviewer (JH) screened 20% of the studies at each stage, with discrepancies resolved through discussion.

### 2.4 Quality assessment

The Mixed Methods Appraisal Tool (MMAT) version 2018 (Hong et al., 2018) was used to assess the methodological quality of the studies. For the purpose of this review, studies were classified into high (>80%), fair (40%–60%), and poor (0%–20%) quality. While no quality threshold was required for inclusion, ratings informed interpretation. To ensure reliability, a second reviewer (JH) independently assessed 20% of the studies.

### 2.5 Data extraction

The studies underwent thorough examination, and relevant data extraction was recorded in an Excel spreadsheet. Ten percent of the data extracted was checked by a second reviewer (JH).

### 2.6 Data transformation

As this systematic review has both quantitative and qualitative data, a convergent integrative approach to mixed‐methods reviews was used (Sandelowski et al., 2006; Stern et al., 2021). Both quantitative and qualitative extracted data were combined using data transformation to fully inform the research question. Quantitative data were converted into qualitative data, that is, qualitized. In this way, quantitative variables were converted into textual descriptive conclusions without using the numerical results. After data transformation, the data were ready for synthesis.

### 2.7 Data synthesis

Thematic synthesis was used (Thomas & Harden, 2008). This is a three‐stage process involving the following: (1) line‐by‐line coding of text; (2) developing descriptive themes; and (3) developing analytical themes.

Extracted data were imported into NVivo (Version 14) for coding. Research findings that were not directly related to the research question were excluded. Relevant participant accounts and author interpretations were line by line coded. Thematic synthesis was conducted by SA, but frequently discussed with the research team.

The study design was guided by the enhancing transparency in reporting the synthesis of qualitative research (ENTREQ) statement (Tong et al., 2012).

## 3 RESULTS

### 3.1 Search yield

**FIGURE 1:**
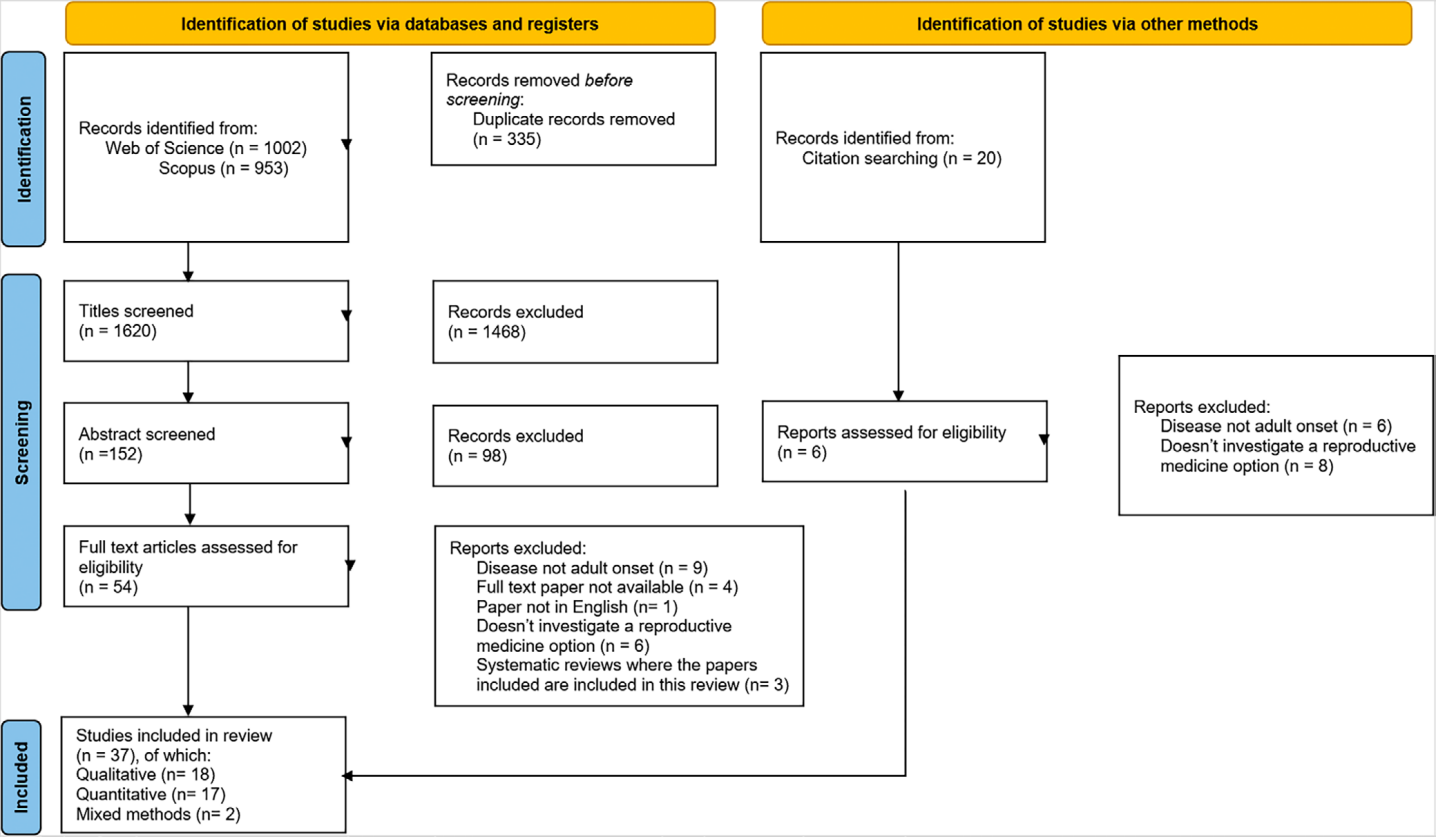
Prisma flow diagram.

The initial search yielded 1620 records, with 1468 excluded by title and 98 by abstract. Of 54 full texts assessed, plus 20 identified via references, 74 were reviewed in total. A total of 37 studies met inclusion criteria, while 37 were excluded. The process is detailed in a PRISMA flow diagram (Figure 1).

### 3.2 Study characteristics

Study characteristics are in Table S1. A total of 17 studies were from Europe, 14 from the United States, three from Australia, two from Israel, and one from Cuba (2003–2024). Sample sizes ranged from 3 to 1081.

Qualitative data were collected in 17 studies via interviews and focus groups and 1 via open questionnaire comments (Quinn, Vadaparampil, King, et al., 2009), while 17 used surveys to collect quantitative data. Qualitative and quantitative data were provided in two studies.

Conditions studied included HBOC (19), Huntington's disease (7), hereditary diffuse gastric cancer (3), inherited heart disease (2), Lynch syndrome (1), melanoma and pancreatic cancer (1), Peutz–Jeghers syndrome (1), familial amyloid polyneuropathy (1), Alzheimer's disease (1), and multiple neurodegenerative conditions (1).

### 3.3 Quality assessment

Most studies were of high quality (mean score 87%), with none below 60%. Three quantitative studies scored 60% due to issues with pretesting questionnaires and sample representativeness. Study quality findings are in Table S2.

### 3.4 Thematic synthesis

Five analytical themes were generated to elucidate attitudes and decision‐making factors regarding reproductive genetic testing in individuals with or at risk of late‐onset genetic diseases. Table S3 outlines each study's contribution to these themes.

#### 3.4.1 Theme 1: Preventing gene transmission

Individuals sought to prevent transmitting the genetic variant to their children, feeling a responsibility to future generations and society to avoid suffering and “eradicate the disease” (Vadaparampil et al., 2009).

##### Genetic responsibility

Individuals often stated that they have a responsibility to use reproductive genetic testing, given the fact that they are aware of the risk and reproductive genetic testing is available to avoid it (Barlevy et al., 2012; Decruyenaere et al., 2007; Derks‐Smeets et al., 2014; Hallowell et al., 2017; Leontini, 2010; Nahshon et al., 2023; Ormondroyd et al., 2012; Quinn, Vadaparampil, King, et al., 2009; Shah et al., 2022; Valdrez et al., 2014; van Rij et al., 2013; Yeates et al., 2022).

It's different when you have a child and something happens to them, but I became aware that I was a carrier or I could pass this on, then I have a responsibility, I felt, and I know that was—the risk was too high. (Yeates et al., 2022, p. 190) (participant affected by inherited heart disease)

Some couples felt a duty toward their child to prevent the transmission (Barlevy et al., 2012; Nahshon et al., 2023; Ormondroyd et al., 2012; Valdrez et al., 2014; van Rij et al., 2013) while other individuals felt a duty for both their potential children and future generations (Yeates et al., 2022). These individuals wanted to prevent the transmission “at any cost” and they believed that any negatives of reproductive genetic testing were outweighed by the health benefits to society by reducing the incidence of the disease.

Couples using reproductive genetic testing felt they “did the right thing” (van Rij et al., 2013), while three studies (Barlevy et al., 2012; Leontini, 2010; Quinn, Vadaparampil, King, et al., 2009) highlighted regrets and guilt over inaction, due to their “failure to act accordingly.”

He felt a lot of guilt eventually especially as we found out more, that he was the one to give it to her, you know what l am saying … we came home and he was like, he wouldn't talk. (Barlevy et al., 2012, p. 32) (individual whose partner is affected by inherited cardiac disease)

##### Avoiding the suffering in a future child

A lot of individuals displayed significant concern about their children inheriting the genetic variant. The desire for a healthy unaffected child and to avoid suffering for their children emerged as one of the most frequently mentioned motivations in support of reproductive genetic testing (Barlevy et al., 2012; Bouchghoul et al., 2016; Dagan et al., 2017; Dean & Rauscher, 2017; Decruyenaere et al., 2007; Dekeuwer & Bateman, 2013; Derks‐Smeets et al., 2014; Dervin et al., 2023; Downing, 2005; Gong et al., 2016; Nahshon et al., 2023; Ormondroyd et al., 2012; Quinn et al., 2010; Quinn, Vadaparampil, King, et al., 2009; Quinn, Vadaparampil, Wilson, et al., 2009; Rubin et al., 2014; Shah et al., 2022; Staton et al., 2008; Tutty et al., 2023; Vadaparampil et al., 2009; Valdrez et al., 2014; van Rij et al., 2013; Yeates et al., 2022). All women in a study by Dagan et al. (2017) wished for a healthy child without the BRCA gene variant.

If I have a daughter, I don't want her to suffer. (Dagan et al., 2017, p. 1073) (participant affected by HBOC)

In this context, people wanted to avoid the suffering on multiple levels: to protect their child from the psychological burden of having the genetic variant (i.e., the risk of the disease) as well as the physical and psychological stress of suffering with the disease (Derks‐Smeets et al., 2014; Ormondroyd et al., 2012; Quinn, Vadaparampil, King, et al., 2009; Tutty et al., 2023; Valdrez et al., 2014; Yeates et al., 2022). Additionally, some expressed the view that watching their child suffer would be worse than personally having the disease (Ormondroyd et al., 2012 ).

This has been impossible, there's no way I'm gonna put my child through that, there's no way (Ormondroyd et al., 2012, p. 7) (participant affected by HBOC)

##### Desire to “wipe out” the disease

A majority of individuals expressed the desire to not only protect their own children, but to completely wipe out the gene variant (Decruyenaere et al., 2007; Dekeuwer & Bateman, 2013; Derks‐Smeets et al., 2014; Hallowell et al., 2017; Klitzman et al., 2007; Nahshon et al., 2023; Ormondroyd et al., 2012; Quinn et al., 2010; Quinn, Vadaparampil, King, et al., 2009; Quinn, Vadaparampil, Wilson, et al., 2009; Vadaparampil et al., 2009; Yeates et al., 2022).

Many saw reproductive genetic testing as the chance to “stop the whole thing…all these genetically carried life or death things could stop overnight with IVF or abortion” (Hallowell et al., 2017, p. 532) (participant affected by Hereditary Diffuse Gastric Cancer). A common reason for supporting PGT was “wishing to obliterate the genetic variant from the world” (Nahshon et al., 2023).

In another study (Ormondroyd et al., 2012), women agreed that despite the lower disease risk in boys with the BRCA gene, the gender of implanted embryos was irrelevant, as the goal was to eliminate the disease. This was further highlighted in Derks‐Smeets et al. (2014), where couples struggled with not being able to avoid male carriers due to PNT only being offered for female fetuses in the Netherlands at the time of the study.

At first we struggled with the fact that in case of a boy no additional diagnostics would be carried out. We preferred a child without BRCA mutation, to put an end to this. (Derks‐Smeets et al., 2014, p. 1109) (participant affected by HBOC)

#### 3.4.2 Theme 2: Finding the threshold: Evaluating the necessity of reproductive genetic testing

This theme highlights the complexity of attitudes toward reproductive genetic testing. While there is general support for their availability, attitudes are influenced by the perceived severity of the disease and personal experiences, with some questioning the technique's necessity.

##### Disease severity

The physical and emotional severity of the disease motivated individuals to consider reproductive genetic testing (Barlevy et al., 2012; Dekeuwer & Bateman, 2013; Derks‐Smeets et al., 2014; Gietel‐Habets et al., 2017; Tutty et al., 2023; van Rij et al., 2013; Yeates et al., 2022) and informed attitudes toward their use.

And why don't they allow it [PGD] here [in France]? Do they think we don't die enough? (Dekeuwer & Bateman, 2013, p. 243) (participant affected by HBOC)

Some individuals with or at risk of HBOC did not deem it severe or burdensome enough to consider reproductive genetic testing (Dekeuwer & Bateman, 2013; Derks‐Smeets et al., 2014; Gietel‐Habets et al., 2017; Ormondroyd et al., 2012; Quinn, Vadaparampil, King, et al., 2009; Rubin et al., 2014).

I would not want to go through the ordeal [of] IVF just for the BRCA gene. (Rubin et al., 2014, p. 162) (participant affected by HBOC)

HBOC was compared to other genetic diseases such as cystic fibrosis, which affects children, and HD, which, despite also being a late‐onset condition, was viewed as more serious due to the lack of treatment. The latter two were viewed as more justified in the use of reproductive genetic testing (Dekeuwer & Bateman, 2013; Ormondroyd et al., 2012; Quinn, Vadaparampil, King, et al., 2009).

This isn't a gene for blindness or cystic fibrosis or some of the other awful genes which actually disable you from birth and have a massive impact on every aspect of your life; it [HBOC] doesn't disadvantage you, it might never disadvantage you. (Ormondroyd et al., 2012, p. 7) (participant affected by HBOC)

Individuals affected by Peutz‐Jeghers syndrome (PJS) (Van Lier et al., 2012) were generally positive about the use of reproductive genetic testing, but acceptance significantly dropped when applied to PJS specifically.

Pregnancy termination was accepted for HD as it was viewed as a serious disease (Klitzman et al., 2007). Pregnancy termination was also largely supported in the study by Pierron et al. (2023) where participants considered both PNT and PGT acceptable for numerous neurodegenerative diseases as they were viewed as severe enough.

##### Necessity of reproductive genetic testing

Participants had varied attitudes regarding the necessity of reproductive genetic testing. There was some opposition to PGT and PNT due to incomplete penetrance (Nahshon et al., 2023; Ormondroyd et al., 2012).

No, definitely no (to TOP for BRCA). It's just that they've got to take precautions against it, so to me it's just not major. It's not like saying “there I've given you cancer” is it? There's only a percentage chance (Ormondroyd et al., 2012, p. 6) (participant affected by HBOC)

Some participants, aware of the disease's clinical variability and its unpredictable effects on future children, found this uncertainty intolerable, leading them to favor the necessity of reproductive genetic testing (Barlevy et al., 2012; Yeates et al., 2022).

You can do all the [clinical] screening in the world…the uncertainty of the clinical pathway, of a child with the gene, not knowing whether they would express it or not. So I just couldn't face a lifetime of that (Yeates et al., 2022, p. 190) (participant affected by Inherited Heart Disease)

Where diseases had a non‐genetic background risk, participants pointed out that using reproductive genetic testing would not guarantee a child would never get the disease, which informed attitudes (Derks‐Smeets et al., 2014 ).

The availability of preventative and treatment options for some diseases (Derks‐Smeets et al., 2014; Quinn, Vadaparampil, King, et al., 2009), and hope for future treatments, underscored the view that reproductive genetic testing might have been unnecessary or might have become unnecessary by the time participants wanted children (Barlevy et al., 2012; Dean & Rauscher, 2017; Decruyenaere et al., 2007; Derks‐Smeets et al., 2014; Hallowell et al., 2017).

These things (breast/ovarian cancer) can be diagnosed and treated early; I consider breast cancer to be curable and controllable. (Quinn, Vadaparampil, King, et al., 2009, p. 445) (participant affected by HBOC)

In contrast, despite some available prevention and treatment options, reproductive genetic testing was deemed necessary to avoid the physical and emotional burdens of treatment and spare others from experiencing these same burdens (Barlevy et al., 2012; Derks‐Smeets et al., 2014 ).

Well, if you classify this as a good preventive measure… when you, as a 27 or 28 year old woman, have to let them amputate your breasts… This I think, you cannot classify as a good measure, that's just nonsense (Derks‐Smeets et al., 2014, p. 1106) (participant affected by HBOC)

##### The impact of disease experience in determining necessity

Personal disease experience, such as witnessing a close relative with the disease, shaped attitudes on reproductive genetic testing (Barlevy et al., 2012; Dagan et al., 2017; Derks‐Smeets et al., 2014; Downing, 2005; Fortuny et al., 2009; Gietel‐Habets et al., 2017; Tutty et al., 2023; Yeates et al., 2022).

I experienced too much pain and death from this [BRCA mutation] carriership and I feared it (Dagan et al., 2017, p. 1075) (participant affected by HBOC)

In addition to family experience, a personal diagnosis of the disease increases the likelihood of utilizing PGT (Fortuny et al., 2009) and those who struggled with disease‐related surgery wished to shield their future children from these experiences (Tutty et al., 2023).

In contrast, for other individuals the presence and impact of the disease did not motivate them to consider reproductive genetic testing (Tutty et al., 2023; Yeates et al., 2022). This typically occurred when they or the family had experienced fewer symptoms or consequences of the disease.

I think if it was worse, as you say, like I think if we were really affected or we've lost of family member, that would be very different, but our experience of it, it made IVF, for me, sound not worth it in terms of the trouble and the risk (Yeates et al., 2022, p. 189) (participant affected by Inherited Heart Disease)

##### The value of life with a genetic condition

The value of a life with the genetic variant was emphasized by some, with a common theme being, “if my parents had chosen otherwise, I wouldn't be here.” The idea that existing individuals would not be here had an emotional impact (Barlevy et al., 2012; Dekeuwer & Bateman, 2013; Klitzman et al., 2007; Nahshon et al., 2023; Ormondroyd et al., 2012; Tutty et al., 2023; Yeates et al., 2022).

If you say that you're not prepared to have children, are you saying that OUR lives are worthless? Because that's almost what you're saying …no more people should be born of people who have our gene. (Hallowell et al., 2017, p. 532) (participant affected by Hereditary Diffuse Gastric Cancer)

Conversely, the impact of cancer was so profound that others questioned whether the cost of never having existed would have been worth paying.

I do feel that my whole life is cancer, and actually I could cry now, it's awful to think you might not (have been born) but then I wouldn't know the difference … with the knowledge that I have I don't know that I would want my child to have the aimless worry that I feel I have. (Ormondroyd et al., 2012, p. 7) (participant affected by HBOC)

#### 3.4.3 Theme 3: Ethical/acceptability considerations

Individuals differed on whether they believed it was ethical to utilize reproductive genetic testing in order to prevent the transmission of a genetic variant.

##### Acceptability

Ethical views on reproductive genetic testing varied by age, gender, and education, with women and highly educated individuals more likely to find them acceptable (Fortuny et al., 2009). Despite noting ethical concerns, studies showed support, with many considering reproductive genetic testing options ethically justifiable, even if they would not use them personally (Bouchghoul et al., 2016; Chan et al., 2017; Dekeuwer & Bateman, 2013; Dervin et al., 2023; Dewanwala et al., 2011; Fortuny et al., 2009; Gietel‐Habets et al., 2017; Julian‐Reynier et al., 2012; Klatte et al., 2024; Marcheco et al., 2003; Menon et al., 2007; Nahshon et al., 2023; Pierron et al., 2023; Quinn, Vadaparampil, Wilson, et al., 2009; Rubin et al., 2014; Shah et al., 2022; Van Lier et al., 2012; Woodson et al., 2014).

There was also a shared belief that reproductive genetic testing should be routinely offered to carriers and be available and accessible for women to utilize. This was grounded in the view that it is their right to know about and access these options (Chan et al., 2017; Dervin et al., 2023; Gong et al., 2016; Quinn, Vadaparampil, King, et al., 2009; Quinn, Vadaparampil, Wilson, et al., 2009; Vadaparampil et al., 2009; van Rij et al., 2013; Woodson et al., 2014).

##### Wariness of eugenics

Among certain participants, PGT was regarded as extreme and a method for creating a “designer baby.” Even among those who were pro‐choice, some expressed reluctance to pursue PGT or PNT due to wariness of eugenics (Dean & Rauscher, 2017; Klitzman et al., 2007). Others challenged that choosing PGT to prevent the transmission of a genetic disease constituted creating a “designer baby” (Dagan et al., 2017; Dekeuwer & Bateman, 2013). The following quote illustrates reproductive genetic testing was perceived by one participant as about ensuring the health of a future child and not about bestowing seemingly desirable traits.

What? Am I choosing a blond child? I choose a healthy child (Dagan et al., 2017, p. 1075) (participant affected by HBOC)

##### Playing god

Concerns around “playing God” were a key ethical consideration in PGT (Quinn et al., 2010; Quinn, Vadaparampil, Wilson, et al., 2009; Rubin et al., 2014; Tutty et al., 2023; Vadaparampil et al., 2009).

I don't wanna play God and pick [embryos] that don't have [a CDH1 PV]… if they grow up into people, they'll still be perfectly good people too. (Tutty et al., 2023, p. 291) (participant affected by Hereditary Diffuse Gastric Cancer)

The concept of “playing God” was raised in Rubin et al. (2014), but it was believed that it was for a very good reason and therefore justifiable.

I still do feel in a little way like it's playing God, but for a very good reason (Rubin et al., 2014, p. 162) (participant affected by HBOC)

##### When does life begin?

There were varied views in relation to when life begins which additionally influenced attitudes toward reproductive genetic testing. The fetus was viewed as a “baby” (Downing, 2005) and as “children” (Derks‐Smeets et al., 2014) by some individuals.

To some people it's a blob of cells. To me it's a baby the minute that it's in ya, you know… Like on a pregnancy test, you know, when it changes colour, it's like a new life (Downing, 2005, p. 230) (participant affected by HD)

Participants had mixed views on whether an embryo constituted a life or not. The disposal of embryos solely based on their genetic variant was viewed as controversial by some (Derks‐Smeets et al., 2014; Hallowell et al., 2017; Klitzman et al., 2007; Nahshon et al., 2023; Ormondroyd et al., 2012; van Rij et al., 2013; Yeates et al., 2022). On the other hand, discarding embryos was not seen as morally problematic by others as they were not viewed as ‘living beings’ (Ormondroyd et al., 2012).

an embryo in its own right can't be called a living being, can it? (Ormondroyd et al., 2012, p. 7) (participant affected by HBOC)

##### Pregnancy termination

Participants had varied attitudes regarding the ethics of pregnancy termination for adult‐onset genetic conditions, with some studies finding widespread acceptability (Julian‐Reynier et al., 2012; Klitzman et al., 2007; Marcheco et al., 2003; Menon et al., 2007; Ormondroyd et al., 2012; Pierron et al., 2023; Van Lier et al., 2012; van Rij et al., 2013) yet termination was unacceptable for some individuals in other studies (Chan et al., 2017; Decruyenaere et al., 2007; Downing, 2005; Hallowell et al., 2017 ). Some individuals had mixed feelings where they were accepting of pregnancy termination in others but not for themselves (Klitzman et al., 2007).

With abortion, I'm definitely pro‐choice, but I personally would have trouble with that…It would feel immoral to me (Klitzman et al., 2007) (participant affected by HD)

#### 3.4.4 Theme 4: External influences in decision‐making

This analysis highlights key external factors influencing and affecting reproductive decision‐making.

##### Family and relationship factors

Three studies reported that the views of family members influenced participants' decisions to utilize reproductive genetic testing (Dean & Rauscher, 2017; Rubin et al., 2014; Yeates et al., 2022).

So my brother was kind of angry with me when I told him that I was, for a few days, [I was] considering not doing PGT, and rolling the dice. And he got kind of angry that I was willing to take that risk. And that kind of made me jump back a little bit (Dean & Rauscher, 2017, p. 1308) (participant affected by HBOC)

Male carriers specifically expressed feelings of guilt and talked about the difficulty of broaching the subject of risk and reproductive testing to partners (Derks‐Smeets et al., 2014; Hallowell et al., 2017).

I would especially regret that I am the source of the evil in this case and you (i.e., the female partner) would have to go through all this hormone misery (Derks‐Smeets et al., 2014, p. 1107) (participant affected by HBOC)

Woodson et al. (2014) found childless women were more likely to consider PGT and PNT than those with children. For women with prior children, rejecting PGT emphasized valuing the lives of existing children who may have inherited the gene (Yeates et al., 2022).

If it had been prior diagnosed to having [child 1] could've been a different outcome with how we did this process but once we've had [child 1] and fallen in love with him, obviously, it was a no brainer to continue having children, to I guess thinking, you continue rolling that dice. (Yeates et al., 2022, p. 190) (participant affected by Inherited Heart Disease)

##### The influence of healthcare professionals

It was reported that some individuals felt that HCPs disapproved of reproductive genetic testing, with a participant in one study reporting being told by a HCP that PGT was unethical and another felt her doctor was disapproving.

He was kind of hesitant to give me names of doctors [specialising in PGD], but I was pretty forceful. (Rubin et al., 2014, p. 161) (participant affected by HBOC)

##### The influence of knowledge and information

Individuals who had higher knowledge and awareness levels of reproductive genetic testing also had higher levels of acceptance toward reproductive genetic testing (Gietel‐Habets et al., 2017; Vadaparampil et al., 2009). Half of the respondents in one study that did not justify the use of PGT and PNT did so due to a lack of knowledge on them (Pierron et al., 2023), indicating the importance of education and awareness in shaping attitudes. Both female (Quinn, Vadaparampil, Wilson, et al., 2009) and male participants (Quinn et al., 2010) also reported low awareness and knowledge of PGT. Notably, respondents who were aware of PNT were unaware that it could be used for BRCA variants (Dervin et al., 2023) which ultimately influenced their decision‐making.

#### 3.4.5 Theme 5: Psychological and practical concerns of reproductive genetic testing

Across the literature, concerns about the psychological and physical impact of reproductive genetic testing were prominent in participants' decision‐making processes.

##### Psychological concerns

Concerns around the psychological impact of PNT influenced the decision‐making process, with studies emphasizing the psychological strain of a pregnancy termination (Dean & Rauscher, 2017; Decruyenaere et al., 2007; Derks‐Smeets et al., 2014; Downing, 2005; Ormondroyd et al., 2012; Valdrez et al., 2014; van Rij et al., 2013).

Another difficulty of PNT included the long period of uncertainty while waiting for the results, as well as the hesitance to become emotionally attached to the pregnancy until the results, in case of termination (Decruyenaere et al., 2007; Derks‐Smeets et al., 2014).

During the first months of each pregnancy, we felt uncertain and numb. We could not be happy or feel attached to the pregnancy until we knew the result of the CVS. We had a long distressing period of waiting for the results of the CVS. (Decruyenaere et al., 2007, p. 457) (participant affected by HD)

PGT was also seen as an emotionally demanding procedure across the literature. The “artificial way” of conceiving and the requirement of having to turn conception into a medical procedure and losing the sense of romance, control, and spontaneity were significant drawbacks (Decruyenaere et al., 2007; Derks‐Smeets et al., 2014; Dervin et al., 2023; Hallowell et al., 2017; Nahshon et al., 2023).

There was a fear of the PGT procedure itself and worry that they would not be able to cope with it (Hallowell et al., 2017; Klitzman et al., 2007; Nahshon et al., 2023).

My sister has witnessed very close‐up how emotionally draining that can be (Klitzman et al., 2007, p. 355) (participant affected by HD)

##### Practical concerns of IVF

The practical and logistical difficulties were a big influence on reproductive decision‐making. Frequent clinic appointments, the long duration of the process, hormone injections, and the “all consuming” day to day effect of PGT played a role in participants' decisions not to pursue PGT (Dagan et al., 2017; Derks‐Smeets et al., 2014; Hallowell et al., 2017; Yeates et al., 2022).

I want children, but then looking into that IVF, the genetic thing that is a total nightmare by the looks of it. (Hallowell et al., 2017, p. 534) (participant affected by Hereditary Diffuse Gastric Cancer)

Another recurrent factor was the low chance of pregnancy by PGT (Dagan et al., 2017; Derks‐Smeets et al., 2014; Menon et al., 2007; Ormondroyd et al., 2012; Yeates et al., 2022).

Some participants needed fertility support irrespective of their genetic status, or had preexisting embryos from fertility preservation, which influenced reproductive decision‐making (Dagan et al., 2017; Yeates et al., 2022). Some believed that if a woman had fertility issues that might result in IVF anyway, then PGT would be more acceptable (Ormondroyd et al., 2012 ) and this was sometimes the main reason to utilize PGT in Dagan et al. (2017).

[The cancer in the family] was not the main reason. For me, I did PGD because I had to undergo IVF (Dagan et al., 2017, p. 1075) (participant affected by HBOC)

The analysis highlighted a “balancing act” for BRCA gene variant carriers, who must navigate cancer risk management alongside using PGT. This is complicated by IVF hormone risks, the need for early risk‐reducing surgeries (e.g., oophorectomy), and the urgency to conceive quickly (Ormondroyd et al., 2012; Rubin et al., 2014).

it's more important for me to be alive and to be still living rather than jeopardise it by having something that could potentially cause the cancer to come back (Ormondroyd et al., 2012, p. 8) (participant affected by HBOC)

##### Financial considerations

Financial limitations also often made PGT inaccessible (depending on the healthcare system) (Barlevy et al., 2012; Dagan et al., 2017; Gong et al., 2016; Klitzman et al., 2007; Nahshon et al., 2023; Yeates et al., 2022).

It is important that people who want to undergo PGD and give birth to a “healthy” [non‐BRCA1/2 carrier] child will have the chance to do so without taking a “mortgage” …. Only those who have some $25,000 [for several IVF‐PGD cycles] [can do it] (Dagan et al., 2017, p. 1076) (participant affected by HBOC)

In other ways, PGT was seen as a potential to reduce future healthcare costs due to not passing on the disease and therefore the associated medical costs (Barlevy et al., 2012; Quinn et al., 2010; Yeates et al., 2022).

you're paying upfront to save yourself a lot of medical expenses in the future, potentially (Yeates et al., 2022, p. 191) (participant affected by Inherited Heart Disease)

## 4 DISCUSSION

This mixed‐methods systematic review presents a thematic synthesis of 34 studies of late‐onset genetic conditions, which have been explored through five themes.

A key finding was the avoidance of suffering and a desire for a healthy child and not wanting to pass on the genetic variant being one of the strongest motivators for considering reproductive genetic testing. This is consistent with previous research (Genoff Garzon et al., 2018; Hughes et al., 2021). In the Netherlands, at the time of Derks‐Smeets et al. (2014) study, only female fetus' were screened for the BRCA gene variant for PND and individuals in the Netherlands disagreed with this policy as they believed the goal was to eradicate the gene and not just reduce the suffering the gene causes in individual women; this specifically highlighted the finding that the goal is to prevent the transmission of the gene and eliminate the disease rather than reduce the risk to an individual child.

The desire for an unaffected child was particularly true for individuals who have experienced the genetic condition. Reproductive decision‐making was therefore found to be informed by “experiential knowledge” of the disease (personally or within the family). Having an intimate and first‐hand understanding of the nature of the disorder and the suffering it can cause may therefore inform positive attitudes toward reproductive genetic testing. Experiential knowledge is being increasingly recognized as an important factor that influences reproductive decision‐making (Boardman, 2014a; Lombardi et al., 2022). This factor was part of the important finding of the debate over the necessity of reproductive genetic testing as well as other factors like the severity and treatment availability.

Our findings highlight conflicting and at times negative views surrounding reproductive genetic testing for late‐onset diseases, particularly for BRCA gene variants, as seen in prior research (Derks‐Smeets et al., 2014; Hughes et al., 2021). However, for diseases like HD, perceived severity of the disease and suffering it can cause appears to outweigh the possibility that people may live a “normal” life before developing symptoms. Severity therefore played a greater role than the late‐onset characteristic in participants' acceptance of reproductive genetic testing, with previous studies showing that individuals with late‐onset conditions reported poorer health, viewed their condition more negatively, and considered a good quality of life unattainable compared to early onset conditions (Boardman & Clark, 2022).

Literature on reproductive decision‐making in early‐onset diseases reveals key differences compared to late‐onset conditions. Individuals with existing children affected by an early onset condition were more likely to consider reproductive genetic testing for subsequent pregnancies, with 57% considering pregnancy termination compared to 30% of those without children (Henneman et al., 2001). In Henneman et al.'s (2001) study, the diagnosis of their child with CF significantly influenced decisions for future pregnancies, as parents wished to avoid having another child with CF. Experiencing their child's suffering played a major role, whereas in late‐onset diseases, the disease's effects had not yet materialized. Parents at risk of transmitting a late‐onset condition to their children therefore make reproductive decisions with a very different frame of reference based on memories of older relatives with the condition, and sometimes their own experiences, rather than witnessing the suffering of existing children.

In studies on α‐ and β‐thalassemias, concerns about PGT focused on its reliability and accuracy, rather than psychological or practical factors (Wah Hui et al., 2002). In this study, women with fertility issues were more accepting of PGT, consistent with findings in our review. Similarly, a study on β‐thalassemia and aneuploidies reported heightened anxiety and stress during pregnancy while awaiting PNT results, aligning with observations from our review (Chamayou et al., 1998).

This review highlighted how valuing a life with a genetic variant influences decisions about reproductive genetic testing. According to the views of some participants included in the review, selecting only non‐carrier embryos implies lives with both the genetic variants and the potential disease that arises from them are devalued, echoing the “expressivist objection,” which argues that preventing such lives sends a hurtful message to those that live with similar traits (Powell et al., 2000). The “expressivist objection” has also mediated reproductive decisions in other studies (Boardman, 2014b; Boardman & Thomas, 2023). However, individuals with opposing views emphasize more on the prevention of the disease rather than the prevention of lives with it.

The concept of genetic responsibility has emerged as a key concept in discussions of genetic risk, and plays a significant role in motivating individuals to act on their genetic risk knowledge (Leefmann et al., 2017). This was exemplified in this review and a main finding of the analysis with individuals believing they have the responsibility to use reproductive genetic testing for their future children and for society.

Complex decision‐making was a cross‐cutting experience that characterizes and defines reproductive genetic testing for late‐onset conditions. Reproductive decision‐making was a big emotional burden in many studies. Factors like the desire for healthy children, perceived disease severity, and genetic responsibility often clashed with the challenges of reproductive genetic testing. Participants frequently faced a painful conflict between the benefits of preventing gene variant transmission and the drawbacks of reproductive genetic testing, sometimes describing it as a Cornelian conflict (Bouchghoul et al., 2016) which refers to a deep moral struggle in which a person must choose between options that each carry undesirable or negative consequences for themselves or others.

In addition to this complexity, there were also comparisons drawn between techniques. Some participants expressed a difference of attitudes between PGT and PNT. There was generally a stronger preference for PGT across the studies examined; however, some studies showed preferences for PNT. This preference for PGT was often grounded in the view that the disappointment after an unsuccessful PGT was lesser than the challenges faced by those receiving a positive result through PNT, who then had to consider termination of the pregnancy. This underscored the perceived moral difference between PGT and PNT. Any preference for PNT was mainly due to the ability to conceive naturally and spontaneity, and the low chance of pregnancy by PGT.

Furthermore, while most studies concentrate on female carriers, we found that male individuals also experienced a wariness of transmitting their genetic variant. Further, male carriers express feelings of guilt as they recognize that it would be the female partner that would have to go through the physical challenges of reproductive genetic testing despite being the “healthy” partner.

The cost of PGT is a factor influencing decision‐making; however, its impact varies by geographical location. Patients in Australia and the USA are particularly affected by the high cost. In contrast, eligible couples in the United Kingdom can access up to three cycles of PGT through the NHS.

A worthy point to mention is that the studies were only conducted in western countries, with the exception of a Cuban‐based study (Marcheco et al., 2003), and therefore there is a gap in the literature where further research is needed in low‐and middle‐income countries where there may be additional complexities in relation to cultural, religious, organizational, and financial factors. These factors may impact decision‐making across western countries; however, additional research would be needed to explore this in more depth.

Distinctions between conditions or their influence on decision‐making were not discussed in this review, as the abundance of HBOC studies compared to others made it unclear whether differences arose from the disease itself or limited research on other disorders. However, despite variation in the types of conditions explored, similar views were seen across them.

### 4.1 Implications for practice

**FIGURE 2:**
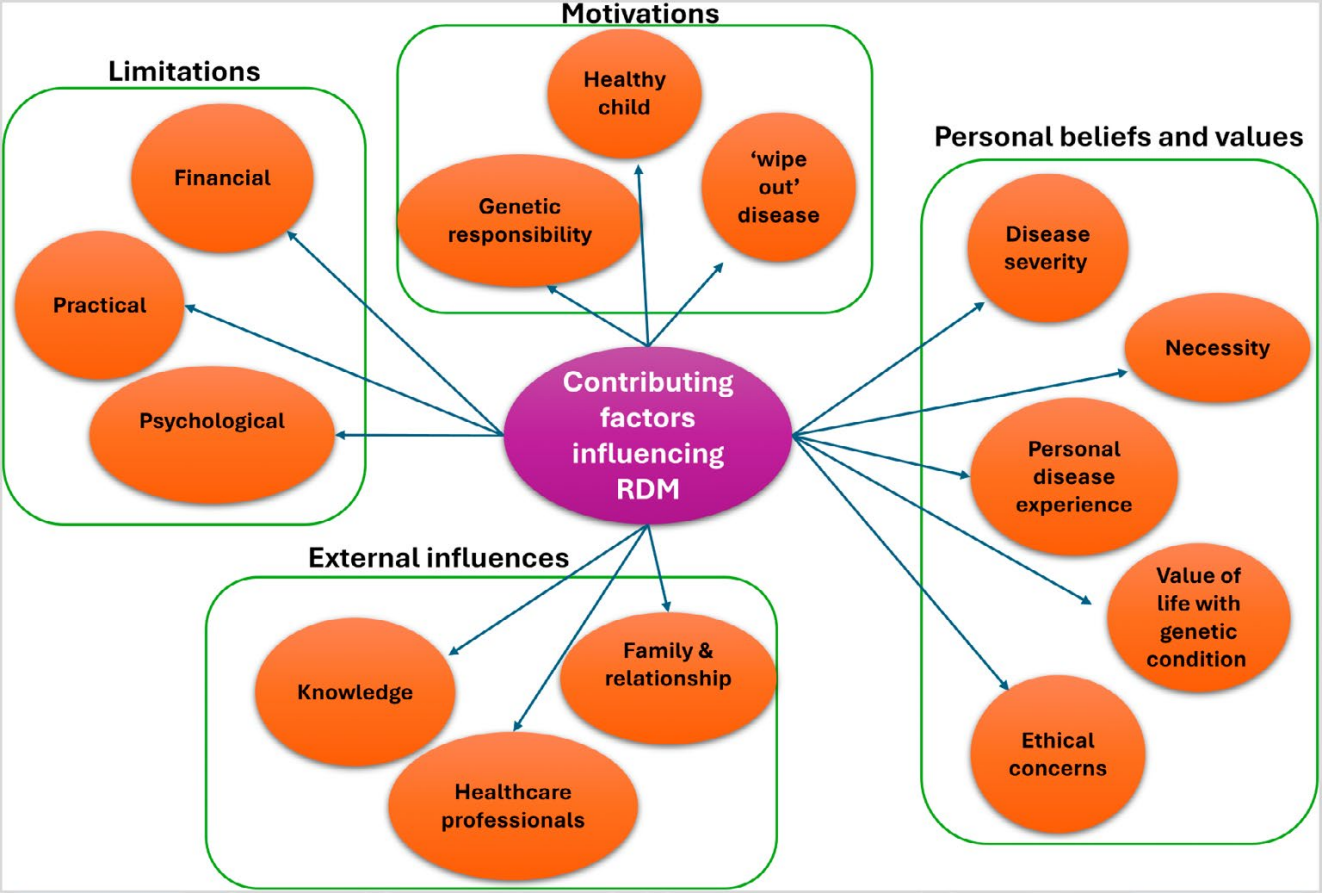
Factors that influence reproductive decision‐making.

The need for awareness and education on reproductive genetic testing is evident, as many studies highlight significant gaps in knowledge among individuals with or at risk of genetic conditions. Future work should look at how to address these knowledge deficits, while exploring individuals' preferences for when and by whom information is delivered. Research shows that greater knowledge correlates with increased acceptability and empowers individuals to make informed decisions, thereby reducing future regret. Healthcare professionals (HCPs) need to be aware of the variety of factors that influence decision‐making. Figure 2 shows the overview of factors highlighted in this review that influence reproductive decision‐making. It is split into the motivations to utilize reproductive genetic testing (Theme 1), individuals personal values and beliefs on reproductive genetic testing (Themes 2 and 3), external influences on attitudes toward reproductive genetic testing (Theme 4), and the limitations of using reproductive genetic testing (Theme 5). Healthcare professionals must understand these factors influencing decision‐making and facilitate value‐aligned, timely discussions that respect individual preferences. Recognizing decision‐making complexity can foster meaningful conversations and address potential challenges.

### 4.2 Limitations

Only two databases were searched, and a specific search string may have limited the results. A further limitation is that distinctions between the different late‐onset diseases are minimally discussed. This is due to the fact that there were significantly more studies on HBOC than other disorders, meaning it was not possible to reliably state that any differences were due to the disease characteristics, rather than a lack of research across the other diseases. However, this is more of a limitation of the existing literature rather than one of the review and suggests future research should focus on a range of adult‐onset genetic conditions, including more rare diseases.

## 5 CONCLUSION

In conclusion, this review highlights the complex interplay of factors influencing attitudes toward reproductive genetic testing, including perceived severity, personal experience with the condition, genetic responsibility, preventing suffering, and what it means to lead a “valuable life.” Varying degrees of personal acceptance were shown, driven by different concerns and beliefs. Nethertheless, individuals generally supported reproductive genetic testing options and believed that they should be available to all. Further research could look at how attitudes change over time, as our results suggest that increased knowledge leads to increased acceptability. Further research could also explore cultural influences on reproductive genetic testing.

## AUTHOR CONTRIBUTIONS

The authors confirm contribution to the paper as follows: Study conception and design: AM, SA; analysis and interpretation of results: SA, JH; draft manuscript preparation: SA; review and edits of draft: JH, AM, CM, FB. All authors reviewed the results and approved the final version of the manuscript.

## FUNDING INFORMATION

SA is funded by a PhD studentship from MND Scotland (2023/MNDS/6400/752McN). CJM is supported by the NIHR Sheffield Biomedical Research Center and an NIHR Research Professorship.

## CONFLICT OF INTEREST STATEMENT

The authors report there are no competing interests to declare.

## Supporting information


Data S1:

## Data Availability

Data sharing is not applicable to this article as no new data were created or analyzed.
