# Supplementary material for: The attitudes of individuals with or at risk of adult‐onset genetic conditions on reproductive genetic testing: A systematic review
Source: J Genet Couns. 2025 Jul 1;34(4):e70079. doi: 10.1002/jgc4.70079 (PMC12210335; doi:10.1002/jgc4.70079)
Supplement: Supplementary file 1 — Data S1: [file JGC4-34-0-s001.docx]

### **Supplementary material**

**Supplementary material 1: Table S1: Study characteristics.**

| **Qualitative studies** | | | | | | |
| --- | --- | --- | --- | --- | --- | --- |
| **Author, Year** | **Country of publication** | **Genetic condition** | **Reproductive option** | **Method used** | **Participants** | **Key conclusions/recommendations of study** |
| Dagan et al., 2017 | Israel | HBOC | PGT | Semi-structured interviews | Ashkenazi Jewish Israeli women, either carriers of BRCA1/2 gene variants or the partners of male carriers | When women and couples embarked on PGD in the hope of securing a healthier future for their offspring, the majority of these were apparently deterred by the complexities that accompany this form of repro-genetic technology, and discontinued the screening procedure even if they had not conceived. The users who eventually opted out of PGD attributed their withdrawal to the emotional burden that PGD entailed, and the concomitant practical difficulties. Further research should be conducted in other PGD clinics in Israel and elsewhere, in order to validate these findings. |
| Dean and Rauscher, 2017 | USA | HBOC | PGT | Semi-structured interviews | Women who received positive BRCA genetic test | Emotions and logic affect family planning decision-making and provide additional information that may assist genetic counselors and other healthcare providers in effectively facilitating conversations about family planning. It is important that during pre-genetic testing counseling sessions genetic counselors discuss reproductive decision-making and possible feelings about passing on one’s genetic variant to children alongside personal risk-reducing health decisions. |
| Rubin et al., 2014 | USA | HBOC | PGT | Semi-structured interviews | BRCA1/2 carriers (male and female) | Narrative case reports may help individuals develop appropriate expectations of PGD for BRCA prepare for possibly challenging decisions and outcomes, and ultimately determine whether it is compatible with their reproductive goals. |
| G.P. Quinn et al., 2009 | USA | HBOC | PGT | Qualitative examination of comments | Women affected by HBOC. | While many women who participated in this study had an unfavorable opinion of the use of PGD for individuals affected by HBOC, some women with a favorable opinion considered PGD as a new option for high risk women who may have previously opted to refrain from having a biological child due to fear of transmitting the genetic variant. The need for educational campaigns to increase PGD awareness and provide information about the procedure, access and affordability, is high within this population. Further research is needed to determine how this population would like this information presented to them. There is also a need to explore attitudes of PGD among health care professionals, including genetic counselors and physicians. |
| Derks-Smeets et al., 2014 | Netherlands | HBOC | PGT & PNT | Semi-structured focus groups and dyadic interviews | Couples carrying a BRCA1/2 gene variant | The perceived severity of HBOC and, for female carriers, the safety of hormonal stimulation and the time frames for PGD planning before preventive surgeries are essential items BRCA couples consider in reproductive decision-making. The emotional impact of this decision should not be underestimated; especially non-users may experience feelings of doubt or guilt up to several years afterwards. PGD counselling with tailored information addressing these items and decisional support in order to guarantee well-informed decision-making is needed. |
| Ormondroyd et al., 2012 | UK | HBOC | PGT & PNT | Semi-structured interviews | Women who received a positive BRCA test before having children | Women had limited awareness of PND/TOP or of PGD for BRCA. Where they were aware, this was mostly as a result of media coverage. Reproductive issues and options are very relevant and appropriate subjects for genetic counselling, particularly for younger carriers who have not begun or completed their families. There is a clear need for additional psychosocial support to help resolve these issues. |
| Dekeuwer and Bateman., 2013 | France | HBOC | PGT | Semi-structured interviews | Carriers of a BRCA1/2 gene variant | Carriers are mainly concerned by the risk of transmitting ‘much more than a gene’: painful experiences not only with respect to health, such as undergoing cancer surveillance or combatting the illness, but also regards to family life, such as witnessing the illness and death of a close relative or reconsidering one’s plans to have a family. As for opinions concerning the acceptability of PGD as a reproductive option, opinions about personal recourse were varied but all expressed the understanding that PGD should be made available to those persons who consider it their best option. |
| Hallowell et al., 2017 | UK | Hereditary Diffuse Gastric Cancer | PGT & PNT | Open-ended interviews | High-risk individuals of HDGC (male and female). Confirmed CDH1 gene variant carriers and/or those who had undergone PTG | Interviewees generally regarded reproductive genetic testing as an acceptable form of HDGC risk management. However, some were concerned that their genetic risks required them to plan reproduction and anticipated difficulties communicating this to reproductive partners. Individuals had a preference for PGD over PND because it avoided the need for a termination of pregnancy. However, those who had not yet had children expressed concerns about having to undergo IVF procedures and worries about their effectiveness and the need for embryo selection in PGD. |
| Tutty et al., 2023 | Australia | Hereditary Diffuse Gastric Cancer | PGT & PNT | Semi-structured interviews | Participants with a positive CDH1 genetic test result | Young people may benefit from re-engagement with genetic services when planning a pregnancy or supporting their children through their own genetic counseling process. A specialized model of longitudinal care that is delivered by genetic counselors trained in youth-friendly practice would be ideal. Findings highlight the unique challenges these young people face when thinking about, planning, and enacting their reproductive goals and performing parental roles whilst managing their increased risk of gastric cancer and offers insights into how to support this patient group. |
| Klatte et al., 2024 | Netherlands | Hereditary cancer (melanoma and pancreatic cancer) | PGT | Online focus group | Individuals with or at risk of a germline CDKN2A pathogenic variant | In conclusion, our results provide insight into a variety of psychosocial aspects regarding genetic testing, skin and pancreatic surveillance in (potential) carriers of a CDKN2A PV. An important reason to undergo genetic testing and participate in surveillance was to gain control over ones' cancer risk. There appeared to be variety in how individuals perceived their risk and experienced burden of surveillance. This warrants further exploration to discern who may benefit from additional psychosocial support. Additionally, we should work toward a centralized source of information covering relevant themes, including cancer surveillance, influence of lifestyle, and family planning. |
| van Rij et al., 2013 | Netherlands | Huntington's disease | ePGT & ePNT | Semi-structured interviews | Dutch couples who intentionally underwent ePND or ePGD | The following topics should be explored during reproductive counselling: the emotional implications of ePND or ePGD, the moral status of the embryo or fetus and values in relation to TOP, the interests of all those involved (future child, at-risk parent, partner), a discussion of all (reproductive) options available, anticipating the future about the possible impact of HD on the future child. Couples carefully consider all moral dilemmas involved, and cope with the considerable emotional strain reasonably well. Candidate couples should receive comprehensive and timely non-directive counselling in respect of all the possible scenarios and adequate professional and psychological support prior to, during and after the test/treatment. |
| Klitzman et al., 2007 | USA | Huntington's disease | PGT & PNT | Semi-structured interviews | Individuals at risk of HD | Providers need to be as aware as possible of how reproductive decisions are viewed and made within the complexities of couple dyads. Health care workers need to be able to raise and address these topics with patients and couples as part of medical care, and learn how to do so. |
| Leontini, 2010 | Australia | Huntington's disease | PGT & PNT | Semi-structured interviews | Individuals at risk of HD | While the message of the meta-narrative on risk is critically interpreted by the informants as the moral expectation to prevent the transmission of HD, through their stories they reframe ethical practice as a process of incorporation, whereby both dominant social values, and the fears, resistances and contradictions these engender, are reworked in the form of counter narratives. |
| Gong et al., 2016 | USA | Huntington's disease | PGT & PNT | Semi-structured, open-ended interviews | HD gene-positive status individuals | This study found that 1) knowing one's gene-positive status results in an urgency to reach milestones of young adulthood and positive changes in young adults’ approach to life; 2) testing positive influences young adults’ education and career choices, romantic relationships, and family planning; and 3) young adults desire flexible and tailored genetic counseling to address needs and concerns unique to this population. They highlight issues unique to the gene-positive pre-symptomatic young adult population that call for further research, intervention and advocacy from professionals within the health and social systems. |
| Downing, 2005 | UK | Huntington's disease | PNT | In-depth interviews | Members of three families facing HD (at risk of HD) | The model of responsibility could inform the “reﬂexive tools” that clinicians employ in genetic counseling sessions and offers clinician a useful checklist of things that matter when negotiating stresses that can arise in families. Identifying these factors would enable clinicians to facilitate decision making that is corresponds with clients’ values and beliefs and acknowledges the impact of the disorder on their lives. Further research is needed to establish whether the model forms a framework in which to compare how those facing other genetic risks and making different decisions negotiate responsibility. |
| Bouchghoul et al., 2016 | France | Huntington's disease | PNT | Semi-structured interviews | Female carriers and spouses of male carriers. | PND was a well-accepted reproductive option for persons at risk of developing HD. Couples requesting PND wish to have a child not at risk for HD, and that women accepted to undergo two or more PNDs to reach this objective. After a second unfavourable result, the desire to have a child was still present, but the burden of the procedure seemed too heavy and they either accept having a child with a 50% risk of carrying the gene variant or they abandoned the idea of having a child. Communication about HD is difficult for families, and parents tend to wait for overt signs of the disease before informing their children; spouses are more proactive than mothers who carry the gene variant. The results should help genetic counsellors present the available reproductive options and their outcome in the face of HD. |
| Yeates et al., 2022 | Australia | Inherited heart disease | PGT | Semi-structured interviews | Individuals with an inherited heart disease and partners | The decision to undergo PGD for inherited heart disease is highly personal and shaped by individual values and experience of disease. Unique to inherited heart disease is the impact of the risk of sudden cardiac death. A road map is provided for discussions with couples contemplating PGD in the setting of inherited heart disease which includes managing expectations of the process of PGD. |
| Barlevy et al., 2012 | USA | Inherited heart disease (LQTS) | Both | Open-ended interviews and focus groups | Individuals who have personal or family histories of cardiac arrhythmia or sudden death | In conclusion, persons who have direct or family experience with LQTS, like people with the experience of many other genetic conditions, have widely varying opinions about reproductive decision making. There is no single way that people with or carriers of the condition feel about reproductive options. There clearly is a need for further research to explore the attitudes of affected individuals toward measures to prevent the birth of a child with a LQTS mutation, including prenatal and preimplantation testing and the use of donor gametes. It would also be valuable to learn how perspectives on reproduction can change over time, with increased knowledge and experience. It will become increasingly important to understand the reproductive impact of genetic diagnosis as laboratory research identifies the genetic bases for a growing number of conditions that raise the risk of sudden death. Such an understanding will be critical to developing informative, noncoercive, and respectful approaches to prenatal counseling and testing. |

| **Quantitative studies** | | | | | | |
| --- | --- | --- | --- | --- | --- | --- |
| **Author, Year** | **Country of publication** | **Genetic condition** | **Reproductive option** | **Method used** | **Participant characteristics** | **Key conclusions/recommendations of study** |
| Chan., et al 2017 | USA | HBOC | PGT & PNT | Online questionnaire | Female BRCA carriers. | Practitioners need to be motivated to incorporate discussions on reproductive issues into their counseling in order to facilitate decision-making on these topics. Information regarding available reproductive options and preimplantation/prenatal diagnosis should be provided, as well as guidance in decision-making for these women. Psychological support could also be offered at the time of testing given the sensitive nature of these topics. |
| Vadaparampil et al., 2009 | USA | HBOC | PGT | Web-based questionnaire | Women at high risk for developing breast or ovarian cancer | Women at increased risk for hereditary cancer may consider preimplantation genetic diagnosis as part of their reproductive decision making. Therefore, it is important to understand existing levels of awareness and attitudes toward this technology to provide optimal counseling and support. |
| Woodson et al., 2014 | USA | HBOC | PGT & PNT | Questionnaire study | Women of childbearing age referred for HBOC evaluation and genetic consultation. | Premenopausal women may benefit from further discussion beyond a one-time genetic result disclosure session. Additional counseling sessions provide an opportunity for improved processing time and repetitive counseling on the important topics and decisions, particularly time to explore the available options, such as cryopreservation and deferring decisions regarding PGD. |
| Gietel-Habets et al., 2017 | Netherlands | HBOC | PGT & PNT | Online cross-sectional survey | Male and female BRCA carriers | Improvement of information provision remains needed, in order to timely inform all couples with HBOC about the available reproductive options and enable them to make a balanced reproductive decision. This may limit the risk of negative psychological impact due to decisional conflict and possible regret. |
| Menon et al., 2007 | UK | HBOC | PGT | Postal survey | Women with BRCA gene variant | The majority of BRCA gene gene variant carriers are supportive of offering PGD to others, thus endorsing the HFEA decision. However, most women would not consider it personally. Concerns raised highlight the need for regular HFEA reviews of the licensing criteria, as HBOC may cease to be a “serious life threatening illness” in the future. |
| Dervin et al., 2023 | France | HBOC | PGT & PNT | Online survey | Women BRCA1/2 gene variant carriers. | BRCA pathogenic variants female carriers do need information about reproductive issues, even if they are not prone to undergo PGT-M nor PND for themselves. |
| G. Quinn et al., 2009 | USA | HBOC | PGT | 33-item quantitative survey | Women with BRCA. | Health care professionals who serve cancer patients should consider incorporating information about PGD into patient education. Further research is needed to survey physicians and genetic counselors about their knowledge and opinions of PGD. |
| Nahshon, Lavie and Oron, 2023 | Israel | HBOC | PGT | Questionnaire | Female BRCA1/2 gene variant carriers. | Despite a substantial proportion of women admitting that genetic variant detection affected their family planning and high acceptance rates, PGT remained exceedingly low. Increasing the knowledge and awareness of these issues is important and should be included in multidisciplinary counselling. Physicians should discuss PGT, family planning and fertility preservation options with BRCA1/2 carriers enabling a personal based decision. |
| Julian-Reynier et al., 2012 | France | HBOC | PGT & PNT | Questionnaire | Unaffected BRCA1/2 gene variant carriers of childbearing age | The closer to reproductive decision-making BRCA1/2 carriers are, i.e., when they are more likely to be making future reproductive plans, the less frequently they intend to have PGD. Carriers' theoretical intentions toward PND are discussed further. |
| Quinn et al., 2010 | USA | HBOC | PGT | Cross-sectional survey | Men who self-reported they were tested for a BRCA gene variant or have a partner or immediate relative who was tested for a BRCA gene variant | High-risk men need more information about PGD and may benefit from educational materials to assist them in reproductive decision-making. High-risk men have limited knowledge about PGD, but do perceive benefits associated with this technology. It is important for genetic counsellors to be sensitive to the needs of high-risk men and incorporate topics related to reproductive decision-making into the genetic counselling session, allowing men to make informed decisions. |
| Staton et al., 2008 | USA | HBOC | PGT | Internet based questionnaire | Female BRCA1/2 gene variant carriers | Young female BRCA1/2 gene variant carriers expressed high concern about hereditary cancer risks to themselves and their children, but relatively low levels of acceptance of currently available assisted reproductive genetic testing including PGD. Future research should further explore the factors driving high-risk women’s choices about reproductive and cancer risk reduction strategies, and should incorporate patient preferences into the development of new interventions to manage hereditary cancer risk. |
| Fortuny et al., 2009 | Spain | HBOC | PGT & PNT | Questionnaire | Male and female candidates for BRCA1/2 genetic testing. | BRCA1/2 genetic results could influence an individual's decisions regarding reproduction. Health care professionals who serve individuals undergoing BRCA testing should incorporate patient education regarding the potential impact of such testing on family planning. Therefore, potential consequences of BRCA1/2 testing on fertility should be fully incorporated and discussed in the genetic counselling process and health care professionals working in this field should inform their patients about available options for not transmitting a genetic susceptibility to cancer. |
| Dewanwala et al., 2011 | USA | Lynch Syndrome | PGT & PNT | Longitudinal questionnaire | Male and female individuals undergoing clinical genetic testing for LS. | Results suggest that a number of men and women at risk for Lynch Syndrome would utilize the information learned from genetic testing in making reproductive decisions. Only a small minority felt that offering prenatal testing for LS would not be ethical. Health care providers should be prepared to discuss the option of assisted reproductive genetic testing during genetic counseling of individuals with hereditary cancer syndromes, such as LS, who are of childbearing age. |
| van Lier et al., 2012 | Netherlands | Peutz-Jeghers syndrome | PGT & PNT | Questionnaire | Adult PJS patients. | The diagnosis of PJS influences decisions regarding family planning in approximately one third of PJS patients, especially in women. Many PJS patients have a positive attitude towards PGD as an option to prevent transmission of PJS to their offspring. In contrast, the attitude of respondents was predominantly negative towards pregnancy termination after PND. Results emphasise the importance of accurate genetic counselling and that medical specialists dealing with patients suffering from hereditary cancer syndromes should discuss aspects regarding family planning, such as PND and PGD. |
| Pierron et al., 2023 | France | Neurological diseases: HD, SCA, CJD, ALS/FTD and MD1 | PGT & PNT | Online questionnaire | Respondents were impacted by autosomal dominant neurological diseases: HD, SCA, CJD, ALS/FTD and MD1. | Self-perceived severity explains the choice of reproductive options very minimally. Medically assisted reproduction is rarely requested by carriers of reproductive age themselves and does not seem to motivate familial communication. The advent of innovative treatments may bring some changes. Genetic counselling should include estimated severity by the testee to better anticipate reproductive behaviour. |
| Valdrez et al., 2014 | Portugal | Familial amyloid polyneuropathy | PGT | Questionnaire | Portuguese FAP carriers (male and female). | Effective educational interventions should focus on the poorer, older, and less educated carriers and on promoting greater conﬁdence in the technique. Taking into account the gene variant carriers’ opinion about ﬁnancial costs and their level of trust in PGD as two key aspects in understanding the options for not using this technique, the corresponding decision-making process should be framed in the public coverage of the treatments and in the public understanding of science and technology. |
| Marcheco et al., 2003 | Cuba | Alzheimer’s disease | PNT | Survey | Fiftysix first-degree relatives of familial cases with AD. | Clear need for education and genetic counseling of family members at high risk, especially for those individuals at risk who are about to make reproductive decisions. |

| **Mixed methods studies** | | | | | | |
| --- | --- | --- | --- | --- | --- | --- |
| **Author, Year** | **Country of publication** | **Genetic condition** | **Reproductive option** | **Method used** | **Participant characteristics** | **Key conclusions/recommendations of study** |
| Decruyenaere at al., 2007 | Belgium | Huntington's disease | PGT & PNT | Quantitative data on reproductive behaviour.  Qualitative: Semi-structured interviews | HD carrier couples | It is clear that reproductive decision-making is a complex process, subject to emotional and unconscious elements. Several factors play a role and some are in conflict, inducing ambivalence. Given these complexities, extensive in-depth counselling is needed, preferably by a counsellor with expertise in genetic testing for HD. Some couples need considerable time to come to reproductive decisions. This means that long-term follow-up counselling is necessary. Counselling should be non-directive and should aim to enhance personal control and free informed decision-making. |
| Shah et al., 2022 | USA | Hereditary cancer (Hereditary Diffuse Gastric Cancer) | PGT | Cross-sectional survey with open- and closed-ended response items | 21 individuals with CDH1 variants | Among patients with CDH1 variants, PGT was identified as acceptable for use in a variety of contexts. Reproductive counseling involving PGT may have benefits that extend beyond CDH1 carriers to help inform or shape their family members’ reproductive behaviors. These findings can help guide providers counseling individuals with CDH1 variants. |

**Supplementary material 2: Table S2: Quality assessment of studies.**

**Key**

| **Yes** |  |
| --- | --- |
| **No** |  |
| **Unclear** |  |

| **Qualitative studies** | | | | | | | | |
| --- | --- | --- | --- | --- | --- | --- | --- | --- |
|  | Screening questions | |  |  |  |  |  |  |
| Studies | Are there clear research questions? | Do the collected data allow to address the research questions? | 1.1. Is the qualitative approach appropriate to answer the research question? | 1.2. Are the qualitative data collection methods adequate to address the research question? | 1.3. Are the findings adequately derived from the data? | 1.4. Is the interpretation of results sufficiently substantiated by data? | 1.5. Is there coherence between qualitative data sources, collection, analysis and interpretation? | Quality score |
| Dagan et al., 2017 |  |  |  |  |  |  |  | 5/5 - 100% |
| Yeates et al., 2022 |  |  |  |  |  |  |  | 5/5 - 100% |
| Dean and Rauscher, 2017 |  |  |  |  |  |  |  | 5/5 - 100% |
| Rubin et al., 2014 |  |  |  |  |  |  |  | 4/5 - 80% |
| Hallowell et al., 2017 |  |  |  |  |  |  |  | 5/5 - 100% |
| G.P. Quinn et al., 2009 |  |  |  |  |  |  |  | 4/5 - 80% |
| van Rij et al., 2013 |  |  |  |  |  |  |  | 5/5 - 100% |
| Derks-Smeets et al., 2014 |  |  |  |  |  |  |  | 5/5 - 100% |
| Ormondroyd et al., 2012 |  |  |  |  |  |  |  | 5/5 - 100% |
| Dekeuwer and Bateman., 2013 |  |  |  |  |  |  |  | 5/5 - 100% |
| Bouchghoul et al., 2016 |  |  |  |  |  |  |  | 5/5 - 100% |
| Tutty et al., 2023 |  |  |  |  |  |  |  | 5/5 - 100% |
| Gong et al., 2016 |  |  |  |  |  |  |  | 5/5 - 100% |
| Klitzman et al., 2007 |  |  |  |  |  |  |  | 5/5 - 100% |
| Leontini, 2010 |  |  |  |  |  |  |  | 4/5 - 80% |
| Downing, 2005 |  |  |  |  |  |  |  | 4/5 - 80% |
| Klatte et al., 2024 |  |  |  |  |  |  |  | 5/5 - 100% |
| Barlevy et al., 2012 |  |  |  |  |  |  |  | 5/5 - 100% |

| **Quantitative studies** | | | | | | | | |
| --- | --- | --- | --- | --- | --- | --- | --- | --- |
|  | Screening questions | |  |  |  |  |  |  |
| Studies | Are there clear research questions? | Do the collected data allow to address the research questions? | 1.1. Is the sampling strategy relevant to address the research question? | 1.2. Is the sample representative of the target population? | 1.3. Are the measurements appropriate? | 1.4. Is the risk of nonresponse bias low? | 1.5. Is the statistical analysis appropriate to answer the research question? | Quality score |
| Dewanwala et al., 2011 |  |  |  |  |  |  |  | 4/5 - 80% |
| Pierron et al., 2023 |  |  |  |  |  |  |  | 3/5 - 60% |
| Chan., et al 2017 |  |  |  |  |  |  |  | 5/5 - 100% |
| Vadaparampil et al., 2009 |  |  |  |  |  |  |  | 4/5 - 80% |
| Woodson et al., 2014 |  |  |  |  |  |  |  | 4/5 - 80% |
| Fortuny et al., 2009 |  |  |  |  |  |  |  | 4/5 - 80% |
| Menon et al., 2007 |  |  |  |  |  |  |  | 3/5 - 60% |
| van Lier et al., 2012 |  |  |  |  |  |  |  | 4/5 - 80% |
| Nahshon, Lavie and Oron, 2023 |  |  |  |  |  |  |  | 4/5 - 80% |
| Gietel-Habets et al., 2017 |  |  |  |  |  |  |  | 4/5 - 80% |
| G. Quinn et al., 2009 |  |  |  |  |  |  |  | 4/5 - 80% |
| Dervin et al., 2023 |  |  |  |  |  |  |  | 4/5 - 80% |
| Valdrez et al., 2014 |  |  |  |  |  |  |  | 4/5 - 80% |
| Quinn et al., 2010 |  |  |  |  |  |  |  | 5/5 - 100% |
| Staton et al., 2008 |  |  |  |  |  |  |  | 4/5 - 80% |
| Julian-Reynier et al., 2012 |  |  |  |  |  |  |  | 4/5 - 80% |
| Marcheco et al., 2003 |  |  |  |  |  |  |  | 3/5 - 60% |

| **Mixed methods studies** | | | | | | | | |
| --- | --- | --- | --- | --- | --- | --- | --- | --- |
|  | Screening questions | |  |  |  |  |  |  |
| Studies | Are there clear research questions? | Do the collected data allow to address the research questions? | 1.1. Is there an adequate rationale for using a mixed methods design to address the research question? | 1.2. Are the different components of the study effectively integrated to answer the research question? | 1.3. Are the outputs of the integration of qualitative and quantitative components adequately interpreted? | 1.4. Are divergences and inconsistencies between quantitative and qualitative results adequately addressed? | 1.5. Do the different components of the study adhere to the quality criteria of each tradition of the methods involved? | Quality score |
| Decruyenaere at al., 2007 |  |  |  |  |  |  |  | 5/5 - 100% (high) |
| Shah et al., 2022 |  |  |  |  |  |  |  | 5/5 - 100% (high) |

**Supplementary material 3: Table S3. The contribution made by each paper to the themes.**

|  | Preventing genetic transmission | | | Finding the threshold | | | | Ethics | External factors that influence decision making | | | Psychological and practical concerns | | |
| --- | --- | --- | --- | --- | --- | --- | --- | --- | --- | --- | --- | --- | --- | --- |
| Study reference | Genetic responsibility | Avoiding the suffering | Wipe out disease | Disease Severity | Necessity of reproductive genetic testing | Disease experience | Life is worthy | Ethical concerns | Family influence | Influence of HCPs | Influence of knowledge | Psychological | Practical concerns of IVF | Financial |
| Chan., et al 2017 |  |  |  |  |  |  |  |  |  |  |  |  |  |  |
| Vadaparampil et al., 2009 |  |  |  |  |  |  |  |  |  |  |  |  |  |  |
| Woodson et al., 2014 |  |  |  |  |  |  |  |  |  |  |  |  |  |  |
| Gietel-Habets et al., 2017 |  |  |  |  |  |  |  |  |  |  |  |  |  |  |
| Menon et al., 2007 |  |  |  |  |  |  |  |  |  |  |  |  |  |  |
| Dervin et al., 2023 |  |  |  |  |  |  |  |  |  |  |  |  |  |  |
| G. Quinn et al., 2009 |  |  |  |  |  |  |  |  |  |  |  |  |  |  |
| Nahshon, Lavie and Oron, 2023 |  |  |  |  |  |  |  |  |  |  |  |  |  |  |
| Julian-Reynier et al., 2012 |  |  |  |  |  |  |  |  |  |  |  |  |  |  |
| Quinn et al., 2010 |  |  |  |  |  |  |  |  |  |  |  |  |  |  |
| Staton et al., 2008 |  |  |  |  |  |  |  |  |  |  |  |  |  |  |
| Fortuny et al., 2009 |  |  |  |  |  |  |  |  |  |  |  |  |  |  |
| Dewanwala et al., 2011 |  |  |  |  |  |  |  |  |  |  |  |  |  |  |
| van Lier et al., 2012 |  |  |  |  |  |  |  |  |  |  |  |  |  |  |
| Pierron et al., 2023 |  |  |  |  |  |  |  |  |  |  |  |  |  |  |
| Valdrez et al., 2014 |  |  |  |  |  |  |  |  |  |  |  |  |  |  |
| Marcheco et al., 2003 |  |  |  |  |  |  |  |  |  |  |  |  |  |  |
| Dagan et al., 2017 |  |  |  |  |  |  |  |  |  |  |  |  |  |  |
| Dean and Rauscher, 2017 |  |  |  |  |  |  |  |  |  |  |  |  |  |  |
| Rubin et al., 2014 |  |  |  |  |  |  |  |  |  |  |  |  |  |  |
| G.P. Quinn et al., 2009 |  |  |  |  |  |  |  |  |  |  |  |  |  |  |
| Derks-Smeets et al., 2014 |  |  |  |  |  |  |  |  |  |  |  |  |  |  |
| Ormondroyd et al., 2012 |  |  |  |  |  |  |  |  |  |  |  |  |  |  |
| Dekeuwer and Bateman., 2013 |  |  |  |  |  |  |  |  |  |  |  |  |  |  |
| Hallowell et al., 2017 |  |  |  |  |  |  |  |  |  |  |  |  |  |  |
| Tutty et al., 2023 |  |  |  |  |  |  |  |  |  |  |  |  |  |  |
| van Rij et al., 2013 |  |  |  |  |  |  |  |  |  |  |  |  |  |  |
| Klitzman et al., 2007 |  |  |  |  |  |  |  |  |  |  |  |  |  |  |
| Leontini, 2010 |  |  |  |  |  |  |  |  |  |  |  |  |  |  |
| Gong et al., 2016 |  |  |  |  |  |  |  |  |  |  |  |  |  |  |
| Downing, 2005 |  |  |  |  |  |  |  |  |  |  |  |  |  |  |
| Bouchghoul et al., 2016 |  |  |  |  |  |  |  |  |  |  |  |  |  |  |
| Yeates et al., 2022 |  |  |  |  |  |  |  |  |  |  |  |  |  |  |
| Decruyenaere at al., 2007 |  |  |  |  |  |  |  |  |  |  |  |  |  |  |
| Shah et al., 2022 |  |  |  |  |  |  |  |  |  |  |  |  |  |  |
| Klatte et al., 2024 |  |  |  |  |  |  |  |  |  |  |  |  |  |  |
| Barlevy et al., 2012 |  |  |  |  |  |  |  |  |  |  |  |  |  |  |

**Supplementary material 4: PRISMA checklist**

| **Section and Topic** | **Item #** | **Checklist item** | **Location where item is reported** |
| --- | --- | --- | --- |
| **TITLE** | | |  |
| Title | 1 | Identify the report as a systematic review. | 1 |
| **ABSTRACT** | | |  |
| Abstract | 2 | See the PRISMA 2020 for Abstracts checklist. | 2 |
| **INTRODUCTION** | | |  |
| Rationale | 3 | Describe the rationale for the review in the context of existing knowledge. | 4 |
| Objectives | 4 | Provide an explicit statement of the objective(s) or question(s) the review addresses. | 4 |
| **METHODS** | | |  |
| Eligibility criteria | 5 | Specify the inclusion and exclusion criteria for the review and how studies were grouped for the syntheses. | 5 (table on 43) |
| Information sources | 6 | Specify all databases, registers, websites, organisations, reference lists and other sources searched or consulted to identify studies. Specify the date when each source was last searched or consulted. | 5 |
| Search strategy | 7 | Present the full search strategies for all databases, registers and websites, including any filters and limits used. | Table on 42 |
| Selection process | 8 | Specify the methods used to decide whether a study met the inclusion criteria of the review, including how many reviewers screened each record and each report retrieved, whether they worked independently, and if applicable, details of automation tools used in the process. | 5 |
| Data collection process | 9 | Specify the methods used to collect data from reports, including how many reviewers collected data from each report, whether they worked independently, any processes for obtaining or confirming data from study investigators, and if applicable, details of automation tools used in the process. | 6 |
| Data items | 10a | List and define all outcomes for which data were sought. Specify whether all results that were compatible with each outcome domain in each study were sought (e.g. for all measures, time points, analyses), and if not, the methods used to decide which results to collect. |  |
|  | 10b | List and define all other variables for which data were sought (e.g. participant and intervention characteristics, funding sources). Describe any assumptions made about any missing or unclear information. |  |
| Study risk of bias assessment | 11 | Specify the methods used to assess risk of bias in the included studies, including details of the tool(s) used, how many reviewers assessed each study and whether they worked independently, and if applicable, details of automation tools used in the process. | SM 2 (page 55) |
| Effect measures | 12 | Specify for each outcome the effect measure(s) (e.g. risk ratio, mean difference) used in the synthesis or presentation of results. | NA |
| Synthesis methods | 13a | Describe the processes used to decide which studies were eligible for each synthesis (e.g. tabulating the study intervention characteristics and comparing against the planned groups for each synthesis (item #5)). |  |
|  | 13b | Describe any methods required to prepare the data for presentation or synthesis, such as handling of missing summary statistics, or data conversions. | 6 |
|  | 13c | Describe any methods used to tabulate or visually display results of individual studies and syntheses. | 6 |
|  | 13d | Describe any methods used to synthesize results and provide a rationale for the choice(s). If meta-analysis was performed, describe the model(s), method(s) to identify the presence and extent of statistical heterogeneity, and software package(s) used. | 6 |
|  | 13e | Describe any methods used to explore possible causes of heterogeneity among study results (e.g. subgroup analysis, meta-regression). | NA |
|  | 13f | Describe any sensitivity analyses conducted to assess robustness of the synthesized results. | NA |
| Reporting bias assessment | 14 | Describe any methods used to assess risk of bias due to missing results in a synthesis (arising from reporting biases). | NA |
| Certainty assessment | 15 | Describe any methods used to assess certainty (or confidence) in the body of evidence for an outcome. | NA |
| **RESULTS** | | |  |
| Study selection | 16a | Describe the results of the search and selection process, from the number of records identified in the search to the number of studies included in the review, ideally using a flow diagram. | 7 |
|  | 16b | Cite studies that might appear to meet the inclusion criteria, but which were excluded, and explain why they were excluded. | NA |
| Study characteristics | 17 | Cite each included study and present its characteristics. | SM 1 (page 44-54) |
| Risk of bias in studies | 18 | Present assessments of risk of bias for each included study. | SM 2 (page 55-64) |
| Results of individual studies | 19 | For all outcomes, present, for each study: (a) summary statistics for each group (where appropriate) and (b) an effect estimate and its precision (e.g. confidence/credible interval), ideally using structured tables or plots. | NA |
| Results of syntheses | 20a | For each synthesis, briefly summarise the characteristics and risk of bias among contributing studies. | 9-24 |
|  | 20b | Present results of all statistical syntheses conducted. If meta-analysis was done, present for each the summary estimate and its precision (e.g. confidence/credible interval) and measures of statistical heterogeneity. If comparing groups, describe the direction of the effect. | NA |
|  | 20c | Present results of all investigations of possible causes of heterogeneity among study results. | NA |
|  | 20d | Present results of all sensitivity analyses conducted to assess the robustness of the synthesized results. | NA |
| Reporting biases | 21 | Present assessments of risk of bias due to missing results (arising from reporting biases) for each synthesis assessed. | NA |
| Certainty of evidence | 22 | Present assessments of certainty (or confidence) in the body of evidence for each outcome assessed. | NA |
| **DISCUSSION** | | |  |
| Discussion | 23a | Provide a general interpretation of the results in the context of other evidence. | 24-28 |
|  | 23b | Discuss any limitations of the evidence included in the review. | 30 |
|  | 23c | Discuss any limitations of the review processes used. | 30 |
|  | 23d | Discuss implications of the results for practice, policy, and future research. | 28-29 |
| **OTHER INFORMATION** | | |  |
| Registration and protocol | 24a | Provide registration information for the review, including register name and registration number, or state that the review was not registered. | 5 |
|  | 24b | Indicate where the review protocol can be accessed, or state that a protocol was not prepared. | 5 |
|  | 24c | Describe and explain any amendments to information provided at registration or in the protocol. |  |
| Support | 25 | Describe sources of financial or non-financial support for the review, and the role of the funders or sponsors in the review. | 1 |
| Competing interests | 26 | Declare any competing interests of review authors. | 1 |
| Availability of data, code and other materials | 27 | Report which of the following are publicly available and where they can be found: template data collection forms; data extracted from included studies; data used for all analyses; analytic code; any other materials used in the review. |  |

*From:*  Page MJ, McKenzie JE, Bossuyt PM, Boutron I, Hoffmann TC, Mulrow CD, et al. The PRISMA 2020 statement: an updated guideline for reporting systematic reviews. BMJ 2021;372:n71. doi: 10.1136/bmj.n71. This work is licensed under CC BY 4.0. To view a copy of this license, visit<https://creativecommons.org/licenses/by/4.0/>
